# Debate: Promoting capabilities for young people's agency in the COVID‐19 outbreak

**DOI:** 10.1111/camh.12409

**Published:** 2020-08-13

**Authors:** Gabriela Pavarini, David Lyreskog, Kiran Manku, Rosemary Musesengwa, Ilina Singh

**Affiliations:** ^1^ Department of Psychiatry and Wellcome Centre for Ethics and Humanities University of Oxford Oxford UK

## Abstract

The COVID‐19 pandemic is having a pervasive effect on young people's mental health and well‐being, giving rise to feelings of deep uncertainty and lack of control. Inspired by Amartya Sen's capabilities framework, we argue that building capacity and creating opportunities for community and civic engagement during this time will help young people gain agency and well‐being. We highlight two key areas for participatory engagement: coproduction of research, and peer‐led interventions. Providing capabilities for young people's agency not only builds personal resilience, but also strengthens the quality of our research, interventions and overall response to the global health crisis.

Anxiety, uncertainty and lack of control are all experiences reported by young people who responded to surveys about the immediate impacts of the COVID‐19 crisis (Holmes et al., 2020). Our own consultations with 14‐ to 25‐year‐olds in Europe and Africa go further, to indicate that young people worry about the health and well‐being of loved ones and are anxious about their own and their families' financial situations. Critically, many young people do not trust their government leaders to make decisions on their behalf during this time, and they experience deep anxieties around the broader impacts of this crisis on communities, countries and the world, as recession and nationalist impulses rise up around them.

At the same time, many young people report having regained a sense of control during the crisis, through community and civic engagement. Global examples of such engagement are growing. Young people in Kenya have initiated ‘Mutual Aid Kenya’ to help vulnerable members of their local communities handle the outbreak, and young people in Cameroon have started a home production of hand sanitisers to be distributed to the population cost‐free (Wickramanayake, 2020). Engagement in civic activities of this kind can be framed as the ‘pursuit of a valued outcome’, which has long‐lasting positive effects on young people's mental health and well‐being (Ballard, Hoyt, & Pachucki, 2019). Indeed, in a global survey, adolescent participants considered participation in design and implementation of health policy, programmes and systems to be the single most important area for investment (Patton et al., 2016). As the COVID‐19 pandemic unfolds, there are important opportunities to support young people’s civic energy and, through this, to support their mental health and well‐being. In what follows, we position young people's agency as a critical capability (Singh, 2017) to be mobilised during COVID‐19, and we highlight two key areas where this potential can be realised: coproduction of research, and peer‐led interventions.

Numerous new research studies have passively involved young people as respondents to COVID‐19 surveys. While such surveys collect important information, they also risk generating ‘research fatigue’, and they do not motivate young people to become active stakeholders in COVID‐19 research studies. Yet, we know that research that involves young people in conceptual stages of design and during execution is more likely to be sustainable over time and to produce relevant and acceptable outcomes and interventions (Pavarini, Lorimer, Manzini, Goundrey‐Smith, & Singh, 2019).

In addition to active participation in research, it is also important to support young people in the design and delivery of peer‐to‐peer education and mental health interventions. Our consultations with adolescents in the UK and across Africa indicate that they are highly motivated to support each other during this crisis: by sharing experiences, exchanging fact‐based information and providing emotional support. Peer‐led well‐being interventions have been shown to promote hope, self‐confidence and feelings of social inclusion in adolescents (Repper & Carter, 2011). Millions of young people already access online support groups daily; however, young people often lack the skills to provide effective help. The need for evidence‐based, accessible peer support platforms for training and online delivery in mental health and well‐being is clear, particularly in the current COVID‐19 scenario where remote support is the new norm. Governments and NGOs, technology designers, young people and researchers should work together to develop and test targeted peer‐to‐peer strategies for the COVID‐19 crisis and beyond.

Active youth engagement in research and intervention not only produces better outcomes, but also hones young people's sense of personal agency. The economist Amartya Sen proposes that agency is the pursuit of an objective that one values and that one has good reason to value; he defines agency as fundamental to well‐being. The achievement of agency is dependent on capabilities; these include personal abilities *and* opportunities to pursue valued objectives (Sen, 1993). During COVID‐19, school closures, the decline in job opportunities and physical isolation have deprived children and adolescents of the educational, social and emotional support systems that would normally provide such opportunities. Given the risks to these systems, it is urgent that creative and innovative ways are found to promote young people's capabilities. Mental health scientists, policymakers and funders can collectively bolster the infrastructure that enables effective civic engagement via research participation: financial resources, participation forums, training and mentorship opportunities, opportunities for leadership, and appropriate safeguarding and evaluation. These interventions should be mutually constitutive and take inspiration from young people's civic energy and motivations.

Activities that increase capacity and create opportunities for meaningful participatory engagement allow young people to discover and express personal agency, and they potentially contribute to resilience and well‐being. However, to promote young people's capabilities, it will be necessary to critically assess the ethical conventions that view young people, and particularly those with mental health challenges – intersected with ‘hard to reach’ and ‘BAME’ designations – as intrinsically vulnerable. It is true that the deep uncertainties that characterise a pandemic place young people in a vulnerable position (Holmes et al., 2020). Assessment of vulnerabilities and consequent protection of welfare are essential to good practice. However, such considerations should not form a priori reasons to conceptualise young people as capable primarily of passive participation in civic engagements, such as COVID‐19‐related research and peer support initiatives. Indeed, promotion of capabilities for diverse groups of young people is arguably a matter of justice: young people encounter numerous systemic barriers to the forms of civic participation that can foster agency and well‐being.

Building young people's ***resilience*** through the COVID‐19 crisis should involve more than tracking their mental health over time, or ‘giving voice’ to their experiences. By supporting capabilities that allow young people to achieve agency in the COVID‐19 crisis response and beyond, we contribute to the development of resilient citizens and strengthen our communities’ responses to future global crises.

## Ethical information

No ethical approval was required for this article.
